# Supplementary material for: Interaction with single-stranded DNA-binding protein modulates Escherichia coli RadD DNA repair activities
Source: J Biol Chem. 2023 May 2;299(6):104773. doi: 10.1016/j.jbc.2023.104773 (PMC10238739; doi:10.1016/j.jbc.2023.104773)
Supplement: Supporting information [file mmc1.docx]

**Interaction with single-stranded DNA-binding protein (SSB) modulates *Escherichia coli* RadD DNA repair activities**

Miguel A. Osorio Garcia, Elizabeth A. Wood, James L. Keck, Michael M. Cox

**Supporting Information**

**Figure S1**


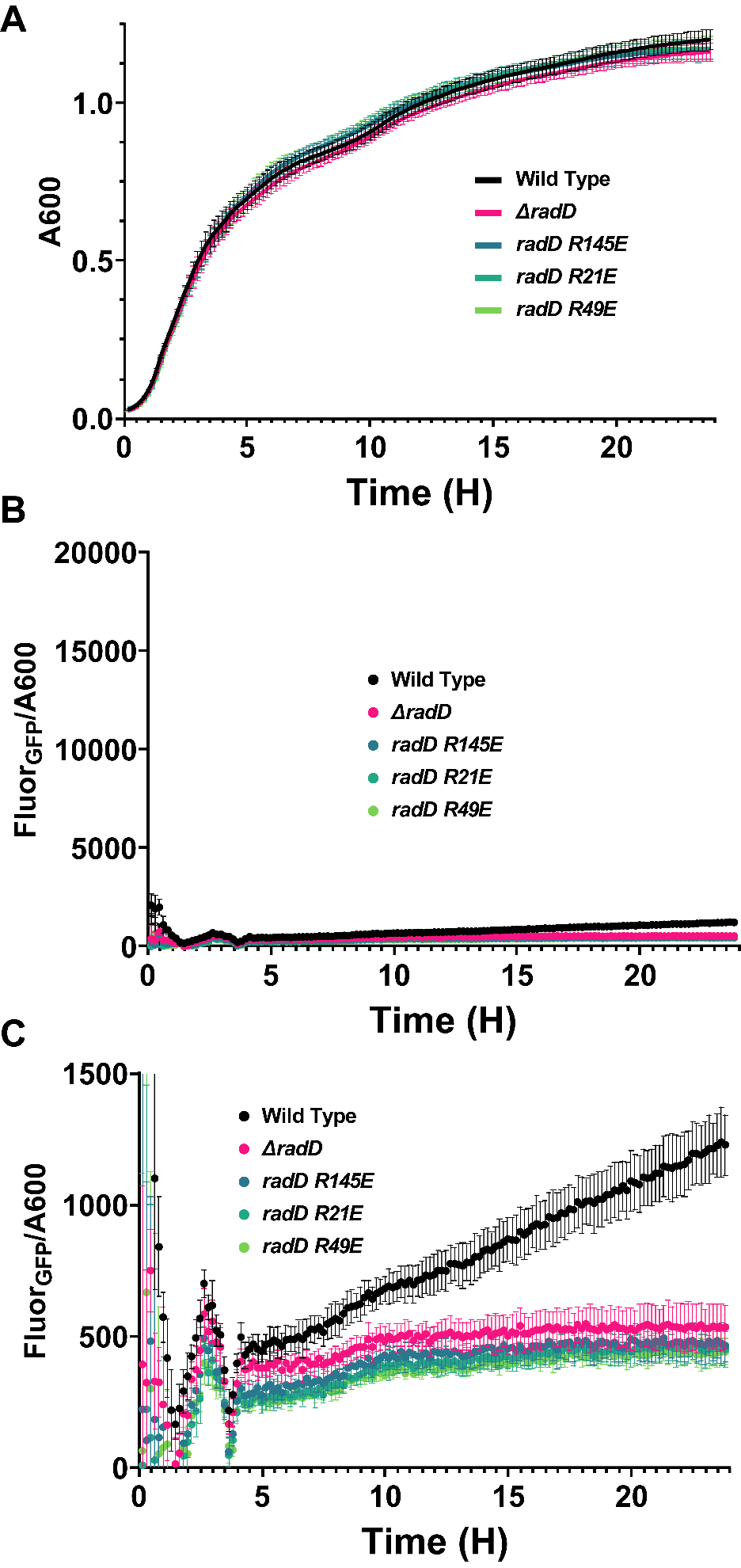


**Figure S1** *SOS induction of radD SSB-binding mutants.* Data points are the mean of 6 independent measurements with error bars representing standard deviation. A) Growth curves of *ΔradD* and SSB-binding *radD* mutant strains compared to wildtype *E. coli*, all transformed with SOS inducible GFP plasmid. B) Normalized SOS induction measured by GFP fluorescence induced by P_recN_ of wildtype, *ΔradD* and radD SSB-binding mutant strains. Using the same Y-axis scale as Figure 4. C) Same data as B with a zoomed in Y-axis scale to highlight differences in the SOS curves.
